# Candida serves as a reservoir associating with facilitating the survival and pathogenesis of *Helicobacter pylori*

**DOI:** 10.3389/fmicb.2026.1774342

**Published:** 2026-02-10

**Authors:** Jianchao Sun, Tingxiu Yang, Qing Luo, Yan Wang, Wei Zhang, Zhenghong Chen

**Affiliations:** 1 Guizhou Key Laboratory of Microbio and Infectious Disease Prevention and Control, School of Basic Medical Science, Guizhou Medical University, Guiyang, Guizhou, China; 2 Key Laboratory of Endemic and Ethnic Diseases, Ministry of Education and Key Laboratory of Environmental Pollution Monitoring and Disease Control, Ministry of Education, Guizhou Medical University, Guiyang, Guizhou, China; 3 Guizhou Provincial Center for Clinical Laboratory, Guiyang, Guizhou, China; 4 Department of Hospital Infection and Management, Guizhou Provincial People’s Hospital, Guiyang, Guizhou, China; 5 The Affiliated Cancer Hospital of Guizhou Medical University, Guiyang, Guizhou, China

**Keywords:** *Candida*, *Helicobacter pylori*, internalization, pathogenesis, survival, urea

## Abstract

Helicobacter pylori (H. pylori) is a key pathogen in gastritis and gastric ulcers, noted for its high recurrence rates. *H. pylori* internalization into *Candida* vacuoles may enhance its survival and pathogenicity. In this study, we investigated the intrinsic association between *H. pylori* and *Candida* by performing *H. pylori*-related assays on *Candida* isolates obtained from clinical fecal samples, gastric mucosal tissues, and vaginal secretions. About 399 isolates of *Candida* were isolated from fecal samples of patients with digestive diseases. *H. pylori* antigens in feces and 16S rDNA within *Candida* isolates were detected, showing a significant correlation. After co-culturing *H. pylori* and *Candida in vitro*, *H. pylori* 16S rDNA-positive *Candida* (CacoHp) were obtained. *In vitro*, the CacoHp strain showed enhanced inhibitory and adhesive effects on GES-1 cells than standard *Candida* strain. *H. pylori* 16S rDNA was detected in CacoHp and *Candida* isolates obtained from gastric mucosal tissue and vaginal secretions. CacoHp and *H. pylori* 16S rDNA-positive clinical isolates exhibited urease activity, as evidenced by the color change of urea containing Sabouraud glucose agar medium from yellow to red. Gene expression analysis indicated that the *H. pylori ureA* gene was expressed at low levels in the CacoHp and *H. pylori* 16S rDNA-positive clinical isolates under the experimental conditions. Using direct immunofluorescence and fluorescence *in situ* hybridization, *H. pylori* antigens and genes were detected in *Candida*. Fluorescent staining indicated the potential presence of viable bacteria within *Candida*, suggesting that viable *H. pylori* might be present intracellularly within these *Candida* cells. These findings suggest that *H. pylori* may persist within *Candida*, with potential implications for human health.

## 1 Introduction

*Helicobacter pylori* (*H. pylori*), a Gram-negative pathogenic bacterium that colonizes the human stomach, has a global prevalence of approximately 50% (Hooi et al., 2017). This bacterium is the primary causative agent of gastritis and peptic ulcers. Notably, 1–10% of individuals infected with *H. pylori* may show progression from chronic active gastritis to severe pathological conditions, including gastric mucosal atrophy, intestinal metaplasia, and development of gastric cancer or mucosa-associated lymphoid tissue (MALT) lymphoma (Yamaoka, 2018).

The eradication of *H. pylori* is essential for the prevention of gastric cancer (Kim et al., 2014). However, between 2000 and 2010, the average eradication rate of *H. pylori* ranged from 80 to 90%, whereas drug-resistant isolates exhibited a significantly lower eradication rate, ranging from 40 to 60% (Glupczynski et al., 2001; Savoldi et al., 2018; Cortés et al., 2021). Hu et al. (2017) analyzed *H. pylori* recurrence rates reported between 1988 and 2017, providing evidence for the association between *H. pylori* recurrence and specific time periods, geographical regions, and socioeconomic statuses. *H. pylori* recurrence is influenced by multiple factors, including treatment regimens, timing and duration of therapy, mode of administration, presence of oral *H. pylori*, formation of the coccoid form, and biofilm development (Sun and Zhang, 2019). Ultimately, recrudescence or reinfection leads to recurrence, representing a major challenge in *H. pylori* management. However, the recurrence of *H. pylori* may be closely associated with its internalization.

*H. pylori* is an obligate extracellular bacterium that primarily colonizes the surface of gastric mucosal epithelial cells, but also has the ability to survive intracellularly—a feature linked to its immune evasion mechanisms and ability to establish persistent infection (Amieva and El-Omar, 2008; Salama et al., 2013). *H. pylori* possesses invasive capabilities in multiple cell types, including gastric mucosal epithelial cells (Wang et al., 2017), macrophages (Du et al., 2016), and HEp-2 cells (a human laryngeal epidermoid carcinoma cell line) (Evans et al., 1992). Notably, clinical isolates obtained from patients in whom eradication therapy failed exhibited significantly higher intracellular invasion efficiency of gastric mucosal epithelial cells than isolates from patients with successful eradication outcomes (Wang et al., 2017). Emerging evidence suggests that *Candida* cells could potentially serve as a reservoir for *H. pylori* transmission while simultaneously providing protection against hostile environmental conditions (Tavakolian et al., 2018; Heydari et al., 2020; Sánchez-Alonzo et al., 2021).

*In vivo*, *Candida* interacts with other members of the human microbiota and encounters co-infecting pathogens. Interactions between *Candida* and symbiotic bacteria, especially *H. pylori*, have developed into complex relationships (Chen et al., 2021; Eichelberger et al., 2023). These interactions occur via direct cellular contact, secretion of signaling molecules or toxins, or modification of the environment to favor one or all interacting partners (De Sordi and Mühlschlegel, 2009; Erb Downward et al., 2013). In this study, we aim to investigate the intrinsic association between *H. pylori* and *Candida* by performing *H. pylori*-related assays on *Candida* isolates obtained from clinical fecal samples, gastric mucosal tissues, and vaginal secretions.

## 2 Materials and methods

### 2.1 Ethics approval and informed consent

All protocols were approved by the Human Medical Ethics Committee of Guizhou Medical University (ethics review approval number: 2022-40). All procedures, including the protocol for obtaining informed consent, were performed in accordance with the principles of the Declaration of Helsinki. Informed consent was obtained from all participants or their legal guardians prior to study initiation.

### 2.2 Strains

The *H. pylori* strain ATCC 700392 (commonly referred to as *H. pylori* 26695) was used. For *Candida*, we used *C. albicans* ATCC 10231 (Ca10231) as well as clinical *Candida* isolates V51 and J115 isolated from vaginal secretions, W49 isolated from gastric biopsy specimens, and F49 from fecal samples. All strains were maintained at the Guizhou Key Laboratory of Microbiology and Infectious Disease Prevention and Control.

### 2.3 Samples

Fecal samples were collected from 399 patients with gastrointestinal disorders at the Department of Gastroenterology of the Affiliated Cancer Hospital, Guizhou Medical University. To diagnose *H. pylori* infection, the colloidal gold method was employed for the detection of *H. pylori* antigen in stool (S_*HpAg*_) (Gisbert et al., 2006). The presence of *H. pylori* antigen in the feces was detected using a Wondfo *H. pylori* Antigen Detection Kit (Guangdong, Wondfo). This kit employs the colloidal gold method to detect *H. pylori* antigen in feces, and is a common method for diagnosing *H. pylori* infection. Fecal samples were used for the isolation and culture of *Candida*. A small quantity of fecal sample was taken and added to sterile physiological saline to prepare a suspension. Subsequently, 0.1 mL of the sample should be added to Sabouraud dextrose agar (SDA, Basebio, Hangzhou, China) containing 50 μg/mL chloramphenicol for the isolation and cultivation of *Candida*. *Candida* was inoculated onto CHROMagar *Candida* medium (CHROMagar, Paris, France) at 37°C for 24–48 for 24 France at 37 e) at 37 *Candida* species were identified based on colony color characteristics: green-blue (*C. albicans*), metallic blue with a pink halo (*C. tropicalis*), mauve (*C. glabrata*), pink and fuzzy (*C. krusei*), and white (other *Candida* species). The isolated *Candida* was stored in 20% glycerol solution at -80°C.

### 2.4 Strain culture

The *H. pylori* 26695 strain was cultured in brain heart infusion (BHI) medium (OXOID, Basingstoke, United Kingdom) supplemented with 7% defibrinated sheep blood (Biological Industries, Henan, China) and incubated under microaerobic conditions at 37°C for 48–72 h. Ca10231 and *Candida* isolates were cultured on SDA supplemented with 50 μg/mL chloramphenicol (Solarbio, Beijing, China). *Candida* cultures were incubated aerobically at 37°C for 24 h.

### 2.5 Co-culture of *H. pylori* and *Candida*

The *H. pylori* 26695 strain and Ca10231 strains were cultured separately under specific microaerobic and aerobic conditions, respectively, at 37°C. Prepare suspensions of the *H. pylori* 26695 strain and Ca10231 strain separately using sterile physiological saline at concentrations corresponding to 5.0 McFarland turbidity and 0.5 McFarland turbidity, respectively. Take 1 mL of *H. pylori* 26695 bacterial suspension and 0.1 mL of Ca10231 suspension, respectively, and add them to 10 mL of BHI liquid medium containing 10% fetal bovine serum. Cultivate under microaerobic conditions at 37 medium 120 r/min for 24 h. Take 100 μL of the post-culture suspension and inoculate it onto SDA medium supplemented with 50 μg/mL chloramphenicol for subsequent cultivation.

### 2.6 Detection of *H. pylori* 16S rDNA in the total DNA extracted from *Candida* cells

*Candida* cells in each experimental group were sub-cultured up to the fifth generation in SDA supplemented with 50 μg/mL chloramphenicol. Subsequently, a specific *H. pylori* gene within each generation of *Candida* was examined following a previously established protocol (Šeligová et al., 2020). DNA used as controls in the polymerase chain reaction (PCR) amplification process was extracted from pure cultures of *H. pylori* 26695 (positive control) and *Ca*10231 (negative control). Briefly, *Candida* cells were suspended in distilled water, and the DAAN nucleic acid extraction or purification reagent (magnetic bead method) was used for DNA extraction from the samples. PCR assays were performed using primers targeting *H. pylori* 16S rDNA to identify *H. pylori* among *Candida*. The primer sequences used for PCR are listed in Table 1.

**TABLE 1:** Primers and the type of PCR reactions employed in this study.

Gene	PCR type	Stage	Primers	Sequence (5’–3’)	Amplicon (bp)
*16S rDNA*	Nested	First	HeliS	AAGAACCTTACCTAGGCTTGACATTG	497
			HeliN	CCGTGGGCAGTAGCCAATT	
		Second	Hpup	TGAGAGAATCCGCTAGAAATAGTGG	454
			Hpdown	TAGCATCCTGACTTAAGGCAAACA	

16S rDNA amplification was performed in a thermal cycler for the first reaction with 94°C for 3 min, 37 cycles of the external amplification reaction (94°C, 45 s; 55°C, 1 min; 72°C, 1 min), 72°C for 5 min, and a hold at 14°C. Use the first-round amplification product as the template for the second round of amplification. The temperature cycle for the second reaction was the same; however, the number of cycles was reduced to 25.

To avoid contamination, negative and blank controls were introduced after every sample in the experiments.

### 2.7 Examination of the ability of *Candida* to adhere to GES-1 cells

The CacoHp strains were continuously passaged up to two times. 1 × 10^6^ GES-1 cells were grafted into a 6-well plate and cultured in serum-free RPMI 1640 medium for 12 h. After the cells adhered, 1 × 10^5^ CacoHp and Ca10231 cells were added. After 24 h of culture, the *Candida* cells adhering to GES-1 cells were counted.

### 2.8 Examination of the toxicity of the *Candida* culture filtrate in GES-1 cells

The CacoHp strains were continuously passaged up to five times. A total of 1 × 10^6^ CacoHp and Ca10231 strains were inoculated into the YPD liquid medium and incubated for 24 h under microaerobic conditions. Following incubation, the cultures of CacoHp and Ca10231 were centrifuged and filtered through a 0.22-μm bacterial filter to obtain sterile culture filtrates. These filtrates were further diluted fivefold with RPMI 1640 medium and co-cultured with GES-1 cells for 24, 48, and 72 h. The cytotoxic effects of the filtrates on GES-1 cells were assessed using a Cell Counting Kit-8 (CCK-8). The cell inhibition rate was calculated as follows: Inhibition Rate (%) = [(Absorbance of Control Group–Absorbance of Experimental Group)/Absorbance of Control Group] × 100%.

### 2.9 Detection of urease activity of *Candida*

In this study, urea-SDA medium was used to detect the urease activity of *Candida*. Phenol red was added to the SDA medium as an indicator, and urea was added as a substrate for the urease reaction. Under slightly alkaline conditions, phenol red turns red, causing the medium to change color from yellow to red. Urea and phenol red were added to the SDA medium to prepare a urea SDA medium containing 5% urea and 0.01% phenol red. *Candida* were inoculated in freshly prepared urea SDA medium and incubated under aerobic conditions at 37 °C for 1–3 days. *H. pylori* 26695 was inoculated onto a urea Columbia serum agar plate (CSA) (5% urea, 10% serum) under microaerobic conditions as a positive control.

### 2.10 Detection of *H. pylori* 16S rRNA by fluorescence *in situ* hybridization

*Candida* strains cultured for 24 h in SDA supplemented with chloramphenicol were removed and washed twice with Phosphate-buffered saline (PBS) (pH 7.4) to ensure cleanliness. Subsequently, the fixed fluid diethyl pyrocarbonate (DEPC) -treated water was immediately added, followed by incubation for a minimum of 12 h. *Candida* samples were dehydrated using a gradient alcohol-paraffin embedding method. A DEPC dilution wash was performed afterwards. Depending on the tissue fixation time, the slices were boiled in a retrieval solution for approximately 10–15 min and allowed to cool naturally. Objective *Candida* cells were marked using a liquid blocker pen based on their characteristics. Proteinase K working solution (20 μg/mL) was applied to cover the objectives and incubated at 37 °C for 15 min. Washing was performed using pure water, followed by three washes with PBS (pH 7.4), each lasting 5 min. Finally, every section was incubated with a pre-hybridization solution at 37 °C for 1 h.

The pre-hybridization solution was removed and replaced with a hybridization solution containing 1 μM of the fungal 18S rRNA probe (5’-CY3-GTGACAAGCATATGACTAC-CY3-3’). The sections were incubated in a humidity chamber at 40°C for overnight hybridization. The hybridization solution was then removed, and the sections were washed in 2 × saline sodium citrate (SSC) for 10 min at 37°C, followed by two washes in 1 × SSC for 5 min each at 37°C, and a final wash in 0.5 × SSC for 10 min at room temperature. Subsequently, the pre-hybridization solution was removed again, and a hybridization solution containing 1 μM of *H. pylori* 16S-1 (5’-FAM-GGAGTATCTGGTATTAATCATCG-FAM-3’) probes was added. The sections were incubated in a humidity chamber and hybridized overnight at 40°C. The hybridization solution was then removed, and the sections were washed in 2 × SSC for 10 min at 37°C, followed by two washes in 1 × SSC for 5 min each at the same temperature, and a final wash in 0.5 × SSC for 10 min at 25°C.

For nuclear counterstaining, the sections were incubated with Nuclei were counterstained with 4’, 6-diamidino-2-phenylindole (DAPI) in the dark for 8 min before mounting. Microscopic examination and imaging were conducted using a fluorescence microscope. DAPI emitted blue fluorescence under ultraviolet (UV) excitation between 330 and 380 nm, with an emission wavelength of 420 nm; FAM emitted green fluorescence under excitation between 465 and 495 nm and an emission wavelength of 515–555 nm; and CY3 emitted red fluorescence upon excitation between 510 and 560 nm, with an emission wavelength of approximately 590 nm.

### 2.11 Detection of *H. pylori* gene expression within *Candida* by quantitative polymerase chain reaction

*Candida* strains isolated from feces, vaginal secretions, and gastric mucosa were cultured on SDA medium for five generations before RNA extraction using the phenol-chloroform method. ABScript III RT Master Mix with gDNA Remover was used to generate complementary DNA (cDNA) from 1 μL of total RNA for qPCR analysis with ChamQ qPCR SYBR Green Master Mix (Vazyme Biotech Co., Ltd. Nanjing, China) on a CFX96 Real-time system (Bio-Rad, Hercules, CA, United States). The primer sequences used for qPCR are listed in Table 2 (Raghwan and Chowdhury, 2014).

**TABLE 2:** Primers used for qPCR.

Gene	Primer	Sequence (5’–3’)	Amplicon (bp)
*ureA*	F	AGCGGTAGCTTTGATTAGTG	268
	R	GCCTTCGTTGATAGTGATGT	
16S rRNA	F	GGGATAGTCAGTCAGGTGTG	245
	R	ACTAGCATCCATCGTTTAGG	

qPCR Testing process was performed in CFX96 Real-time system with 95°C for 30 s, 40 cycles of the external amplification reaction [95°C, 10 s; 60°C, 30 s (Collect fluorescence signals)]. The test results are described qualitatively.

### 2.12 Detection of fluorescent antibodies against *H. pylori* within *Candida*

*Candida* was cultured in the BHI medium (OXOID) at 37°C with shaking at 120 r/min for 24 h. The *Candida* bacterial fluid was centrifuged and washed thrice with saline to obtain a *Candida* bacterial suspension with a McFarland standard concentration of 2. Fluorescein isothiocyanate (FITC)-labeled anti-*H. pylori* IgG antibodies (Cat. No. PA1-73161; Thermo Fisher Scientific) were reconstituted at a dilution of 1:100 to ensure thorough mixing, and the mixture was incubated in a light-protected environment at 25°C for 1 h. A total of 10 μL of the suspension was applied to a glass microscope slide, covered with a coverslip, and examined under a fluorescence microscope at 1,000 × magnification. Ca10231 was used as a negative control.

### 2.13 Detection of bacterial activity within *Candida*

*Candida* isolates cultured on SDA supplemented with chloramphenicol for 24 h were used to prepare *Candida* suspensions with a McFarland standard concentration of 2. A total of 2 μL of LIVE/DEAD^®^ BacLight*™* Bacterial Viability Kits (Thermo Fisher Scientific) was added to 1 mL of *Candida* bacterial suspension and incubated for 15 min at 25°C away from light. A total of 10 μL of the suspension was applied to a glass microscope slide, covered with a coverslip, and examined under a fluorescence microscope at 1,000 × magnification. Ca10231 was used as a negative control.

### 2.14 Data statistics

Data analysis was conducted using GraphPad Prism 9.5.0 software. Measured data were presented as the mean ± standard deviation, and one-way analysis of variance (ANOVA) was employed for comparisons between two groups. Correlation analysis was conducted using the Point-Biserial correlation coefficient. When *P* < 0.05, the difference was deemed statistically significant.

## 3 Results

### 3.1 Examination of *H. pylori* 16S rDNA in *Candida* strains isolated from feces

#### 3.1.1 Detection of *H. pylori* antigen in stool samples

Among the 399 stool samples from which *Candida* was isolated, 112 samples tested positive for S_HpAg_, confirming *H. pylori* infection in these patients.

#### 3.1.2 *Candida* isolation and identification

To explore the relationship between *Candida* and *H. pylori*, we isolated *Candida* species from fecal samples of patients with gastrointestinal disorders and performed species identification. In total, 399 *Candida* isolates were isolated from 997 fecal samples. The distribution of these *Candida* species was as follows: 319 strains were identified as *Candida albicans*, 44 as *Candida krusei*, 14 as *Candida glabrata*, 7 as *Candida tropicalis*, and 15 as other *Candida* species.

#### 3.1.3 Detection of *H. pylori* 16S rDNA in *Candida*

Among 399 isolates of *Candida* isolated from the fecal samples, *H. pylori* 16S rDNA was detected in 93 isolates. To investigate the correlation between *H. pylori* infection in patients and *H. pylori* 16S rDNA-positive *Candida*, the Point-Biserial correlation coefficient was used to analyze the correlation between fecal *H. pylori* antigen and *Candida* isolates with *H. pylori* 16S rDNA. The findings demonstrated a significant correlation between fecal *H. pylori* antigen and *H. pylori* 16S rDNA-positive *Candida* (*P* < 0.001), indicating that the presence of *H. pylori* 16S rDNA in *Candida* was associated with *H. pylori* infection (Table 3).

**TABLE 3:** Detection of *H. pylori* antigen in feces and *H. pylori* 16S rDNA in *Candida* strains.

*H. pylori* infection	*H. pylori* 16S rDNA		Total
	+	-	
S_HpAg_	+ 49	63	112
	- 44	243	287
Total	93	306	399

### 3.2 Acquisition and characteristics of *H. pylori* 16S rDNA-positive *Candida* strains

#### 3.2.1 Acquisition of *H. pylori* 16S rDNA*-positive Candida* strains

CacoHp strains were sub-cultured consecutively for five passages. Following *Candida* genomic DNA extraction, PCR testing for *H. pylori* was performed. The results of the PCR test demonstrated positivity for *H. pylori* 16S rDNA. Consistent findings were also observed in the isolates obtained from partial gastric mucosa, fecal samples, and vaginal secretions.

#### 3.2.2 CacoHp strain exhibited stronger adhesion capability toward GES-1 cells

**FIGURE 1:**
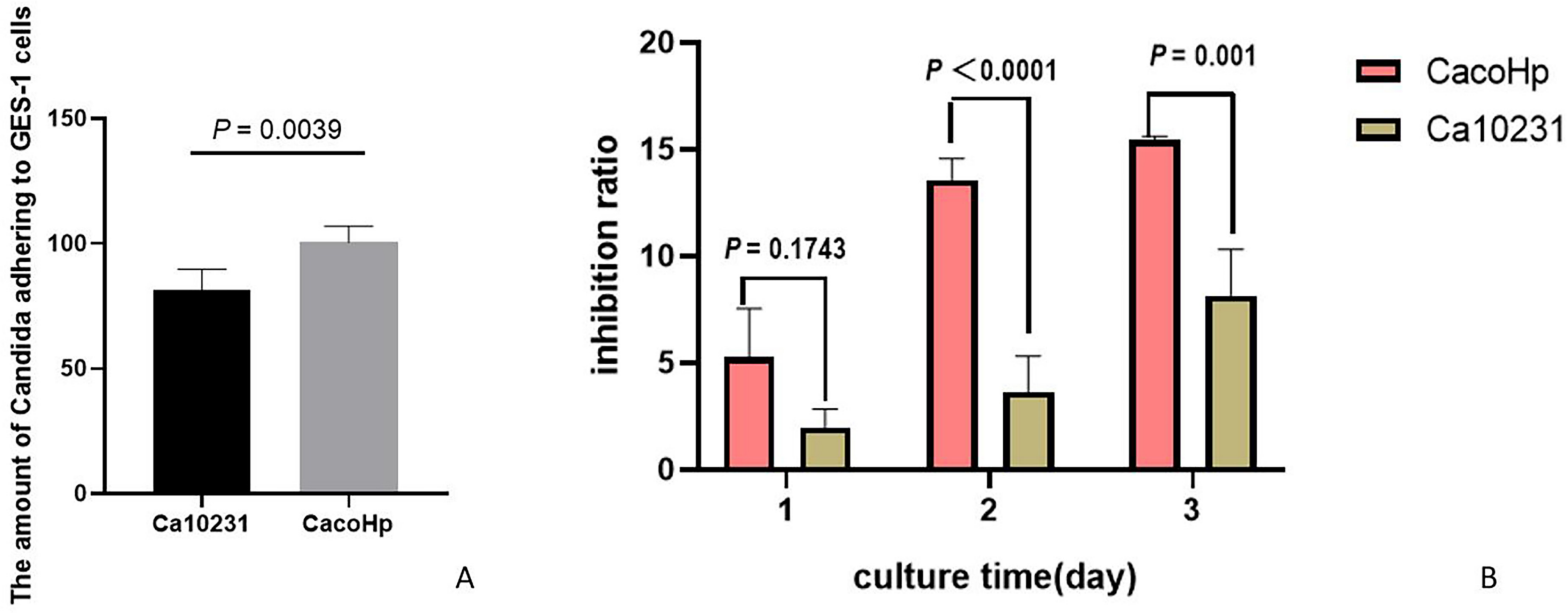
Ca10231: *Candida* standard strain ATCC10231. CacoHp: Laboratory-prepared *Candida* (ATCC 10231) internalized by *H. pylori* (ATCC 700392). **(A)** Adhesion effect of *Candida* on GES-1 cells. **(B)** Inhibitory effect of *Candida* on proliferation toward GES-1 cells.

To investigate the effect of co-culturing *H. pylori* with *Candida* on the adhesion capacity of *Candida* to GES-1 cells, we selected second-generation CacoHp cells. The number of CacoHp strains that adhered to GES-1 cells was significantly higher than that of the Ca10231 strain (Figure 1A). These results indicate that CacoHp exhibits enhanced adhesive properties toward GES-1 cells, which may be associated with the colonization of *H. pylori* 16S rDNA-positive *Candida* strains in the gastrointestinal environment.

#### 3.2.3 CacoHp strain exhibited a stronger inhibitory effect on proliferation toward GES-1 cells

To investigate the inhibitory effect of co-culturing *H. pylori* with *Candida* on proliferation toward GES-1 cells, fifth-generation CacoHp was selected. The results demonstrated that CacoHp exhibited a significantly greater inhibitory effect on GES-1 cells compared to Ca10231 (Figure 1B). This inhibitory effect on proliferation increased in a time-dependent manner and became progressively more pronounced. These findings suggest that *H. pylori* 16S rDNA-positive *Candida* strains exert continuous toxic effects on GES-1 cells.

The adhesion and colonization of bacteria on the cell surface represent critical steps in bacterial pathogenicity (Huang et al., 2016). This study demonstrated that CacoHp cells exhibit significantly stronger adhesion to GES-1 cells. These findings may explain why *Candida* can be detected in the stomachs of patients with gastric ulcers and gastritis. Furthermore, this study provides insights into the mechanism by which *H. pylori* 16S rDNA-positive *Candida* colonizes the stomach.

### 3.3 Detection of *H. pylori*-related characteristics in H. pylori 16S rDNA-positive *Candida* isolated from clinical specimens

#### 3.3.1 *Candida* exhibited urease activity

**FIGURE 2:**
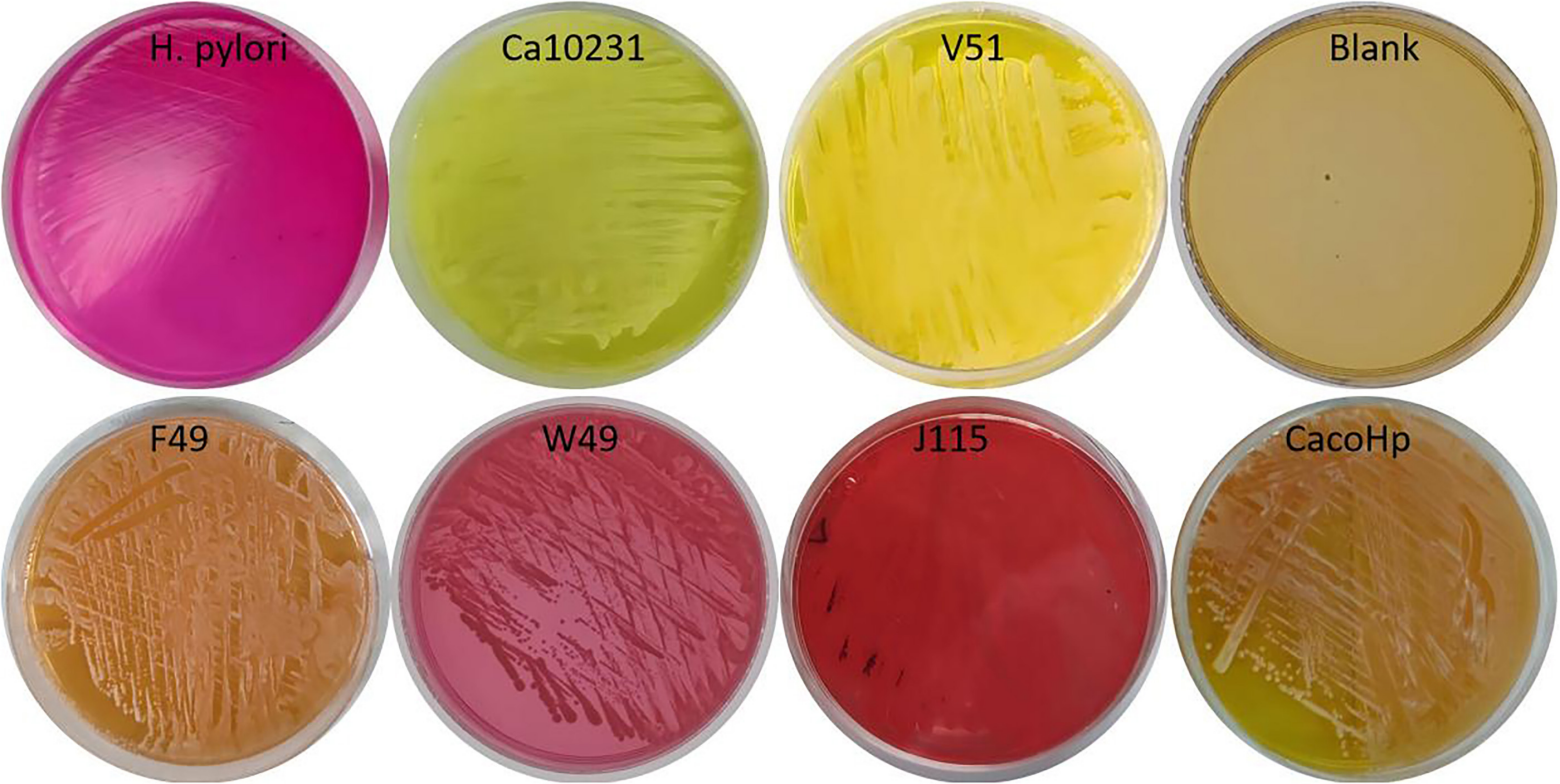
Ca10231: *Candida* standard strain. V51: *H. pylori* 16S rDNA-negative *Candida* isolated from vaginal secretions. F49, W49, J115: *Candida* strains isolated from feces, gastric mucosal tissue, and vaginal secretions, respectively. CacoHp: Laboratory prepared *Candida* internalized by *H. pylori*. The F49, W49, J115, and CacoHp resulted in a noticeable transition of the medium color from yellow to red, similar to *H. pylori*. Conversely, no discernible color change was observed in Ca10231, V51 or in the blank control.

After 3 days of incubation in urea-SDA medium, the W49, F49, and J115 *Candida* strains induced a color change in the medium from yellow to red. This phenomenon may be attributed to the metabolic production of urease by internalized *H. pylori*, resulting in an elevated pH and subsequent color change (Figure 2).

As *Candida* does not contain the *ureA* gene and cannot express urease, the ability of *H. pylori* 16S rDNA-positive *Candida* to induce a color change in urea-SDA medium suggests that urease production originates from *H. pylori*. As *H. pylori* can metabolize urea to produce large amounts of urease, *Candida* strains harboring *H. pylori* 16S rDNA may secrete urease derived from *H. pylori*, thereby generating ammonia and increasing the pH of the medium. This pH shift causes phenol red to transition from yellow to red under alkaline conditions. The production of urease by *H. pylori* 16S rDNA-positive *Candida* during its metabolic processes further suggests that these strains possess stronger pathogenic potential than other *Candida* strains.

#### 3.3.2 Detection of *H. pylori* 16S rRNA in *Candida* strains

**FIGURE 3:**
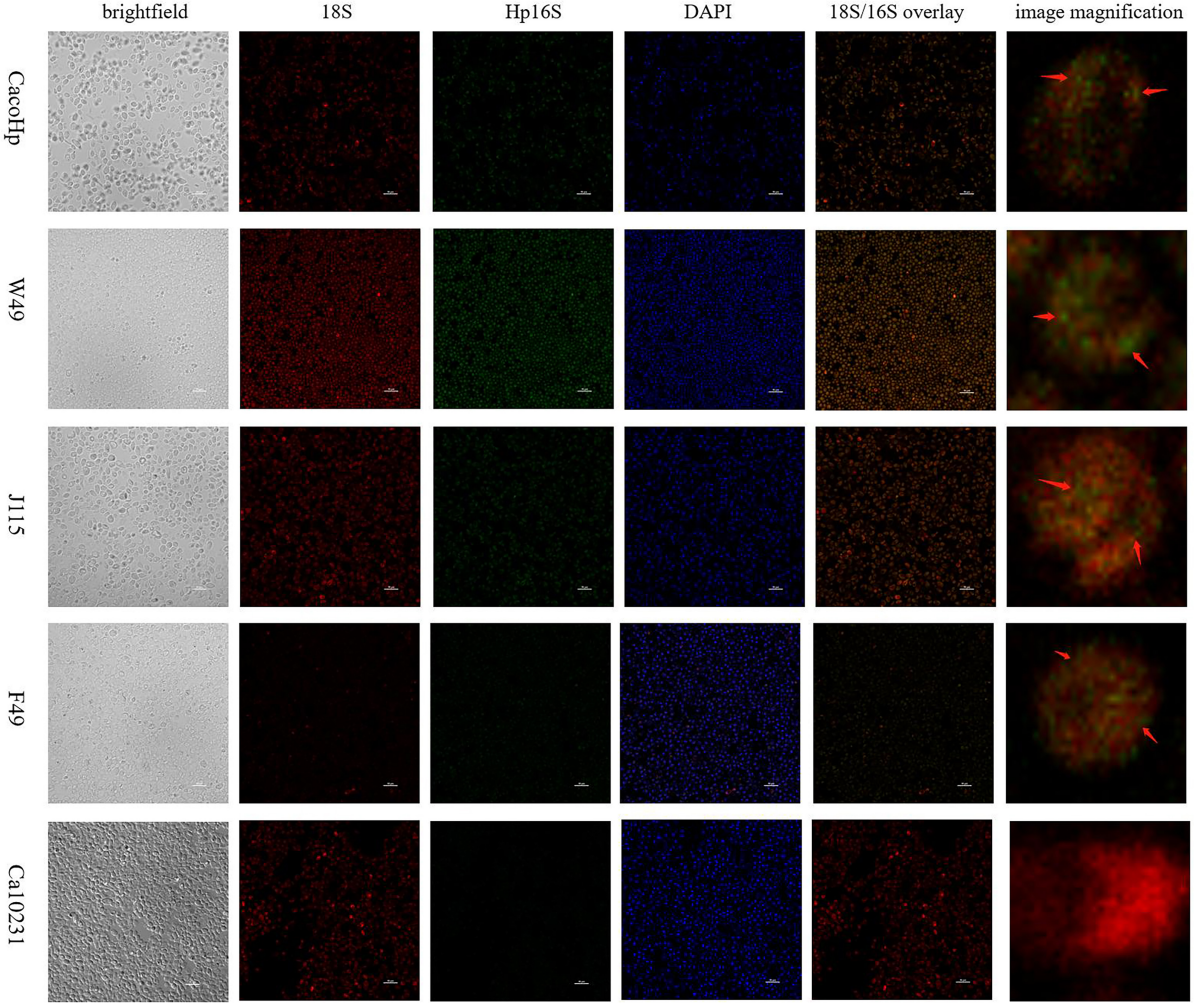
FISH staining with *H. pylori*-specific probes in *Candida* strains. Fungal ribosomes were stained with a universal 18S rRNA probe (red), and *H. pylori* was detected using a specific 16S rRNA probe set (green). Co-localization of 18S and 16S fluorescence (red arrowheads) indicates the presence of *H. pylori* 16S rRNA in *Candida* cells. Scale bars = 10 μm.

To further investigate whether *H. pylori* 16S rRNA is present in *Candida*, we designed an *H. pylori*-specific 16S rRNA probe and observed a similar signal in CacoHp, F49, W49, and J115 strains, all of which are *H. pylori* 16S rDNA-positive *Candida* isolates and green fluorescent spots can be observed within the cells of *Candida* (Figure 3).

#### 3.3.3 Detection of *H. pylori* 16S rRNA and *ureA* gene expression in *Candida* strains

Using FISH, we identified *H. pylori* 16S rRNA in *Candida*. Results obtained from the urea SDA medium further suggested that the urease gene of *H. pylori* may be expressed in *Candida*. Therefore, we employed qPCR to detect both *H. pylori* 16S rRNA and the *ureA* gene in *Candida*. These strains may express *H. pylori* 16S rRNA and the *ureA* gene. The positive results indicate that *Candida*, once internalized by *H. pylori*, can potentially maintain stable expression of *H. pylori* genes. The results are summarized in Table 4.

**TABLE 4:** Results of *H. pylori* gene expression within *Candida* assessed by qPCR.

*Candida* Strain	16S rRNA (Cq)	Result	*ureA* (Cq)	Result
Ca10231	/	Negative	/	Negative
V51	/	Negative	/	Negative
W49	32.29 ± 2.38	Positive	33.83 ± 1.84	Positive
J115	30.67 ± 2.62	Positive	34.20 ± 1.77	Positive
F49	32.03 ± 5.23	Positive	32.44 ± 1.91	Positive
CacoHp	29.41 ± 3.68	Positive	34.62 ± 2.04	Positive
Blank	/	Negative	/	Negative
*H. pylori*	17.36 ± 1.33	Positive	18.78 ± 2.51	Positive

Ca10231: *Candida* standard strain. V51: *H. pylori* 16S rDNA negative *Candida* isolated from vaginal secretions. F49, W49, and J115: *Candida* strains isolated from feces, gastric mucosal tissue, and vaginal secretions, respectively. Caco-Hp: Laboratory preparation of *Candida* that internalized *H. pylori. H. pylori: H. pylori* 26695. Cq: cycle threshold. /: Not detected. The *Candida* strain that internalized *H. pylori* expressed low levels of *H. pylori* gene expression. The Cq value is a key indicator in qPCR experiments for quantifying target nucleic acids, representing the number of cycles it takes for the fluorescence signal to reach a set threshold.

### 3.4 Detection of antigens against *H. pylori* within *Candida* strains

**FIGURE 4:**
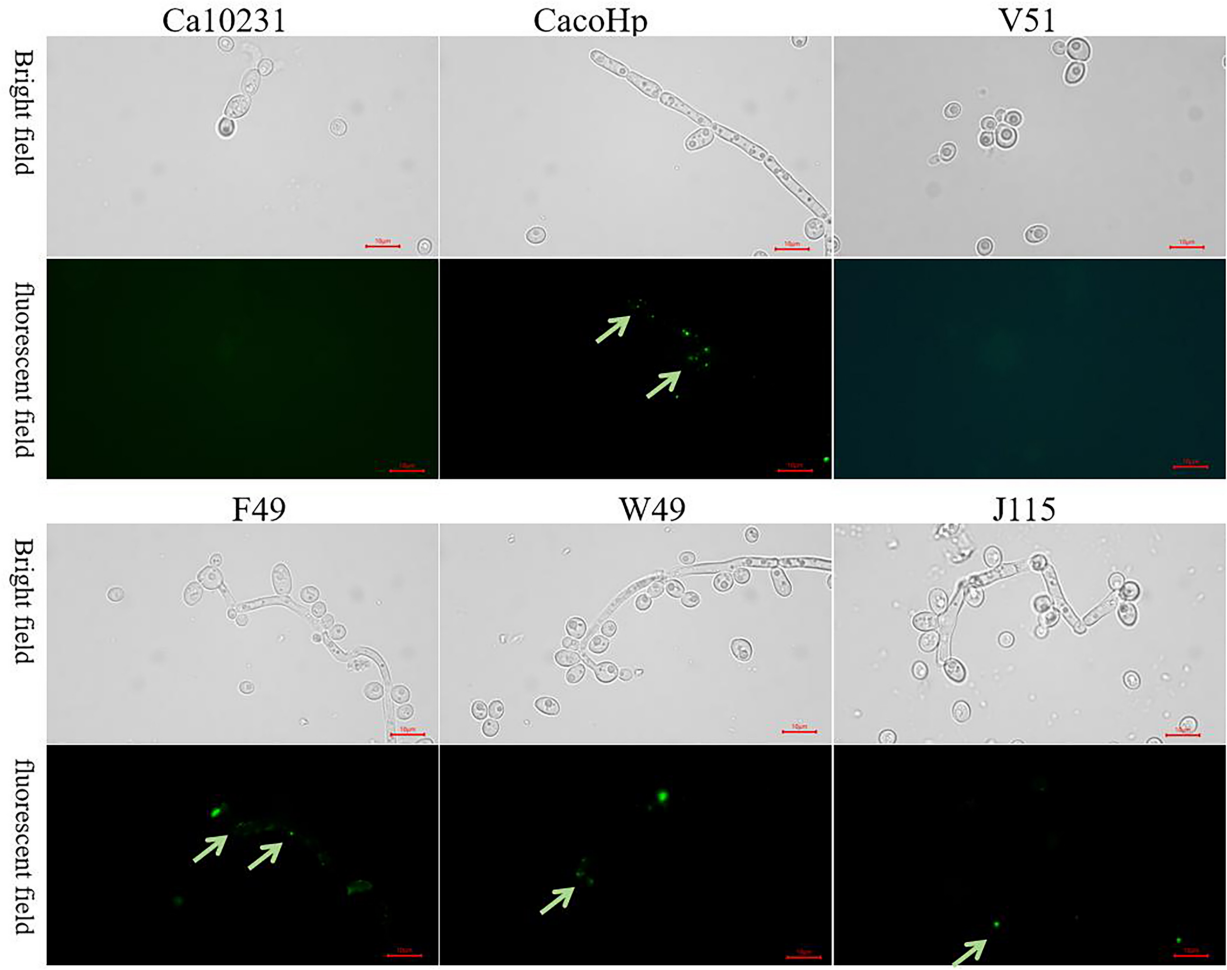
Ca10231: *Candida* standard strain. V51: *H. pylori* 16S rDNA-negative *Candida* isolated from vaginal secretions. F49, W49, J115: *Candida* strains isolated from feces, gastric mucosal tissue, and vaginal secretions, respectively. CacoHp: Laboratory prepared *Candida* internalized by *H. pylori*. No green fluorescence was detected in the *Candida* standard strain, and no *H. pylori* antigen was present. F49, W49, J115, and CacoHp exhibited distinct green fluorescence (green arrow), indicating specific binding of FITC-labeled anti-*H. pylori* IgG antibodies to *H. pylori* antigen inside *Candida* cells. Magnification = × 1,000.

The FISH and PCR results indicated the presence of *H. pylori* nucleic acids in *H. pylori* 16S rDNA-positive *Candida*. To further confirm the presence of *H. pylori* in *Candida*, we employed a direct immunofluorescence assay to detect *H. pylori* antigens within *Candida* cells. Under a fluorescence microscope, no green fluorescence was observed in the Ca10231 strain. However, *H. pylori* 16S rDNA-positive *Candida* exhibited distinct green fluorescence, indicating specific binding of FITC-labeled anti-*H. pylori* IgG antibodies with *H. pylori* antigens inside *Candida* cells (Figure 4). Therefore, the presence of *H. pylori* antigens was confirmed in *H. pylori* 16S rDNA-positive *Candida* cells.

### 3.5 Viable bacteria may exist within *Candida* strains

**FIGURE 5:**
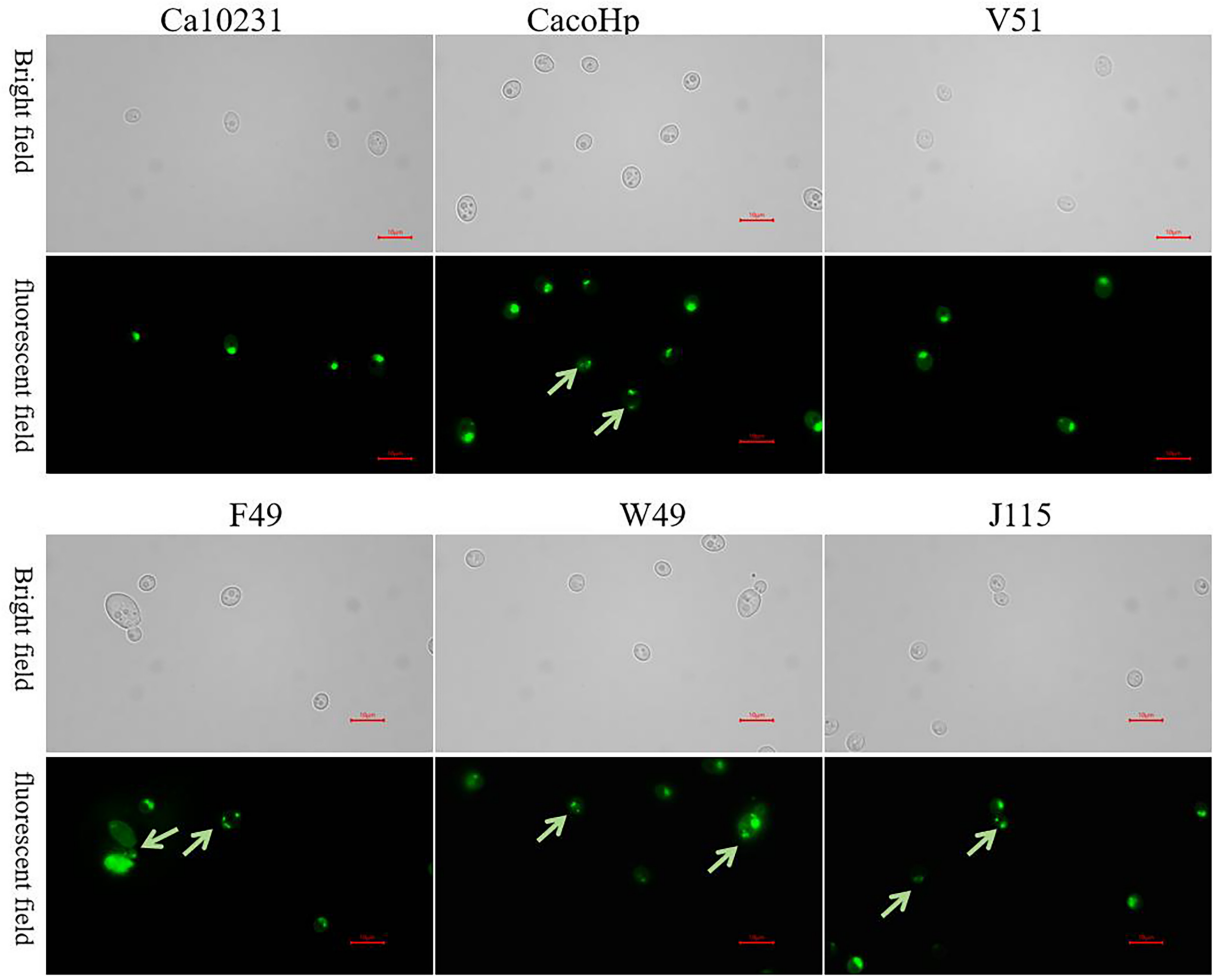
Ca10231: *Candida* standard strain. V51: *H. pylori* 16S rDNA negative *Candida* isolated from vaginal secretions. F49, W49, J115: *Candida* strains from feces, gastric mucosal tissue, and vaginal secretions, respectively. CacoHp: Laboratory-prepared *Candida* internalized by *H. pylori*. No green fluorescence was observed in the *Candida* standard strain, F49, W49, J115, and CacoHp exhibited faint green fluorescence (green arrow). The results suggest that *H. pylori* 16S rDNA-positive *Candida* may contain viable *H. pylori* bacteria.

The LIVE/DEAD^®^ BacLight Bacterial Viability Kit was used to assess bacterial cell membrane integrity. Bacteria with intact membranes fluoresced green, indicating metabolic activity, while those with compromised membranes, suggestive of cell death, fluoresced red. Green fluorescent signals were observed within *H. pylori* 16S rDNA-positive *Candida*, indicating the presence of *H. pylori* with intact membranes and the potential viability of *H. pylori* within *Candida* (Figure 5).

## 4 Discussion

*H. pylori* is a gram-negative pathogen that colonizes the human stomach in specific gaseous environments and nutritional requirements. *H. pylori* exhibit abundant urease production, along with oxidase and catalase activities, as well as metabolism and generation of multiple virulence factors crucial for colonization and pathogenicity (Clyne and Ó Cróinín, 2025). *H. pylori* have been classified as a class I carcinogen due to its clear pathogenic effect.

Infection with *H. pylori* is strongly associated with an increased risk of gastric cancer. *H. pylori* infection can cause chronic gastritis and peptic ulcers. Chronic inflammation induces sustained damage to the gastric mucosal cells and establishes a microenvironment that facilitates gene mutations, potentially promoting tumorigenesis. The *H. pylori* Kyoto Global Consensus (2015) (Sugano et al., 2015) and the *H. pylori* Maastricht V/Florence Consensus (2016) (Malfertheiner et al., 2017) both assert that *H. pylori* infection is closely associated with the development of gastric cancer, and eradication of *H. pylori* is an effective preventive measure against gastric cancer. However, the therapeutic outcomes of *H. pylori* treatment remain unsatisfactory. The primary factor contributing to the ineffectiveness of *H. pylori* eradication is the growing resistance to commonly prescribed antibiotics (McNicholl et al., 2012; Gong and Yuan, 2018; Chey et al., 2024). Additionally, the invasion of *H. pylori* into the vacuoles of *Candida* strains may be associated with recurrence (Yang et al., 2024).

It is now well-established that diverse symbiotic fungal communities exist both internally and externally on mammalian surfaces (Alexiev et al., 2023; Limon et al., 2017). The emerging concept of a fungal bacteriome refers to bacteria residing within and closely associated with fungal host cells (Robinson et al., 2021). Evidence suggests co-colonization of the upper gastrointestinal tract by both *H. pylori* and *Candida*, suggesting a significant association between *Candida* and *H. pylori* in the ulcerative lesions of gastric ulcers, non-ulcer dyspepsia, and giant gastric ulcers (Ramaswamy et al., 2007). *Candida* isolates obtained from clinical fecal samples showed *H. pylori* 16S rDNA with a positivity rate of 28.1%. A correlation was observed between the detection of *H. pylori* 16S rDNA *Candida* and fecal *H. pylori* antigen test results in patients. Clinical sample test results suggest a potential interaction between *Candida* and *H. pylori*, which may exacerbate disease severity or contribute to chronic infections.

To verify the adhesion and toxicity of *H. pylori* 16S rDNA-positive *Candida* toward GES-1 cells, we co-cultured *Candida* with *H. pylori in vitro* to obtain *H. pylori* 16S rDNA-positive *Candida*. *In vitro* cell experiments demonstrated that the CacoHp strain filtrate exhibited significantly stronger adherence and inhibitory effects in GES-1 cells. The enhanced toxicity of CacoHp may be associated with the presence of *H. pylori* in *Candida* cells. This further explains why *Candida* can be detected in patients with gastritis or gastric ulcers.

While *Candida* usually does not colonize the stomach, we found that *H. pylori* 16S rDNA positive *Candida* exhibited a significantly stronger adhesion capability toward GES-1 *in vitro*. Furthermore, the detection of *H. pylori* 16S rDNA in *Candida* isolates suggests the internalization of *H. pylori* into *Candida*. *H. pylori* can enter *Candida* vesicles, where it produces toxic metabolites (Heydari et al., 2020). In this study, the isolated *Candida* exhibited urease activity, which is not a common biological characteristic of *Candida* but represents a prominent feature and pathogenic mechanism of *H. pylori*. The presence of *H. pylori* 16S rDNA-positive *Candida* in the stomach may be associated with positive urea activity test results.

Although recent research has started elucidating the mechanisms underlying bacterial-fungal interactions in opportunistic infections (MacAlpine and Lionakis, 2024; d’Enfert, 2009), our understanding of how these interactions contribute to pathogenesis remains largely incomplete. Notably, we observed the internalization of *H. pylori* into *Candida* cells present in the gastric mucosa, as well as intestinal and vaginal isolates of *Candida*. This suggests a potential mechanism for the transmission of pathogenic *H. pylori* via internalization into *Candida* cells (Yang et al., 2023). Therefore, the interaction between *H. pylori* and *Candida* may represent a novel pathogenic mechanism associated with *H. pylori* infection.

Taking together, the results of the direct immunofluorescence, fluorescence *in situ* hybridization, and bacterial activity detection, it is possible that *H. pylori* may exist as a complete bacterial entity within *Candida* cells, maintaining a relatively low level of metabolic activity. *H. pylori* may reside in *Candida* cells in the form of coccoid bodies. Consequently, during *in vitro* isolation and culture processes, *H. pylori* within *Candida* was not isolated. However, under the influence of environmental factors, nutritional conditions, and *in vivo* cytokines, live *H. pylori* are released from *Candida* cells.

Recent studies have demonstrated the role of fungi in maintaining bacterial microbiota balance and influencing overall intestinal health (Krüger et al., 2019). However, the mechanism by which *Candida* affects bacterial colonization remains unclear. In our study, the internalization of *H. pylori* by *Candida* conferred the ability to break down urea, a property not observed in standard *Candida* strains. qPCR successfully identified gene expression of *H. pylori*. These findings provide compelling evidence for the interplay between *H. pylori* and *Candida*, suggesting that internalized *H. pylori* can maintain gene expression within *Candida*.

Furthermore, the internalized state of *H. pylori* within *Candida* may enable its persistence as an active entity while benefiting from the conducive environment provided by *Candida* under specific conditions. Consequently, this internalized form of *Candida* harboring *H. pylori* has the potential to establish chronic infections or trigger recurrence when external conditions change. Understanding the mechanisms underlying the interplay between *H. pylori* and *Candida* opens new avenues for research on chronic infections and potential treatment strategies. By targeting this internalized form of *Candida* harboring *H. pylori*, it may be possible to develop more effective therapies to disrupt symbiotic relationships and prevent recurrent infections.

These findings provide insights into a potential novel mechanism of pathogenesis involving *Candida* or *H. pylori* alone or in combination. However, we were unable to isolate cultivable *H. pylori* from *Candida*. Further investigations are necessary to elucidate the precise processes by which internalization occurs. Our research has not yet conclusively demonstrated that *H. pylori* can persist for an extended period of time within *Candida* cells while maintaining its structural integrity and metabolic activity. Whether *H. pylori* can remain as an independent, structurally complete, and viable bacterium within *Candida* cells remains to be further investigated. Moreover, it would be valuable to explore whether viable forms of *H. pylori* could be released from internalized *Candida* cells into the surrounding environment. Understanding this aspect may have significant implications for disease transmission and treatment strategies. Future research should adopt a multidisciplinary approach, integrating molecular biology techniques with genomics, chemical, and microbial ecology methodologies, biophysical investigations, and ecological modeling to comprehensively examine the interactions between *Candida* and *H. pylori*.

## 5 Conclusion

Overall, our study provides important insights into the complex relationship between *Candida* and *H. pylori*. *Candida* may provide a protective microenvironment against bactericidal agents, which could, which could, in part, explain cases of treatment failure and recurrence. Moreover, after internalization by *Candida*, *H. pylori* may retain its metabolic activity and with the potential continue to produce toxic metabolites, leading to chronic damage to the gastric mucosa. These insights may help guide the exploration of new preventive or therapeutic approaches.

## Data Availability

The original contributions presented in this study are included in this article/supplementary material, further inquiries can be directed to the corresponding author.
